# Atrial Fibrillation and Fibrosis: Beyond the Cardiomyocyte Centric View

**DOI:** 10.1155/2015/798768

**Published:** 2015-07-01

**Authors:** Michele Miragoli, Alexey V. Glukhov

**Affiliations:** ^1^Centre of Excellence for Toxicological Research, Department of Clinical and Experimental Medicine, University of Parma, Via Gramsci 14, 43126 Parma, Italy; ^2^Humanitas Clinical and Research Centre, Via Manzoni 56, Rozzano, 20090 Milan, Italy; ^3^Department of Cardiovascular Sciences, National Heart and Lung Institute, Imperial Centre for Translational and Experimental Medicine, Hammersmith Campus, Imperial College London, Du Cane Road, London W12 0NN, UK

## Abstract

Atrial fibrillation (AF) associated with fibrosis is characterized by the appearance of interstitial myofibroblasts. These cells are responsible for the uncontrolled deposition of the extracellular matrix, which pathologically separate cardiomyocyte bundles. The enhanced fibrosis is thought to contribute to arrhythmias “indirectly” because a collagenous septum is a passive substrate for propagation, resulting in impulse conduction block and/or zigzag conduction. However, the emerging results demonstrate that myofibroblasts* in vitro* also promote arrhythmogenesis due to direct implications upon cardiomyocyte electrophysiology. This electrical interference may be considered beneficial as it resolves any conduction blocks; however, the passive properties of myofibroblasts might cause a delay in impulse propagation, thus promoting AF due to discontinuous slow conduction. Moreover, low-polarized myofibroblasts reduce, via cell-density dependence, the fast driving inward current for cardiac impulse conduction, therefore resulting in arrhythmogenic uniformly slow propagation. Critically, the subsequent reduction in cardiomyocytes resting membrane potential* in vitro* significantly increases the likelihood of ectopic activity. Myofibroblast densities and the degree of coupling at cellular border zones also impact upon this likelihood. By considering future* in vivo* studies, which identify myofibroblasts “per se” as a novel targets for cardiac arrhythmias, this review aims to describe the implications of noncardiomyocyte view in the context of AF.

## 1. Introduction

The normal function of the heart is a painstaking cooperation between cardiomyocytes and fibroblasts. It is well known that cardiomyocytes provide the “pumping” function of the organ, whereas fibroblasts are responsible for organizing the cellular scaffold and maintaining the proper 3D-network and thus the normal mechanical function. Moreover, fibroblasts contribute importantly to the uniformity of the excitable substrate and to the continuous and rapid electrical activation of the myocardium. In the healthy normal heart, fibrosis-related arrhythmia is normally absent, which indicates that although fibroblasts outnumber cardiomyocytes roughly three-to-one [1], they do not exert any arrhythmogenic effect. Though there is a general assumption that cardiomyocytes play the crucial role in atrial arrhythmogenesis, little is known regarding an “active” role of the connective tissue in this respect.

A variety of pathological conditions, including pressure overload, volume overload, infarction, and aging [2], induces structural remodelling of the heart leading to heart failure and cardiac arrhythmias. This structural remodelling involves changes in the 3D organization of the heart and is based on complex and diverse responses to injury; as a result, all types of cardiac cells are involved. Histopathologically, cardiac remodelling typically involves changes in myocytes size (hypertrophy), the activation and proliferation of fibroblasts, uncontrolled deposition of the extra cellular matrix (ECM), and cell death [3]. This is in favour of the beginning and perpetuation of supraventricular and ventricular arrhythmias due to the presence of collagenous septa, which physically separate regions of cardiomyocytes, thus inducing structural discontinuities at cellular and tissue levels. This can result in conduction block and zigzag conduction, both of which permit structurally determined reentrant propagation of cardiac impulse.

Functionally, cardiac remodelling leads to mechanical dysfunction which increases the likelihood of life-threatening cardiac arrhythmias [10]. Consequently, arrhythmias arising from structurally remodelled hearts are caused by changes in electrical properties of cardiomyocytes and/or by the remodelling of the ECM.

Electrically, remodelling of cardiomyocytes affects a large number of ion channels, ionic pumps, and proteins [11, 12]. Furthermore, redistribution and regulation of gap junction proteins (connexins) alter the physiological anisotropic ratio, which causes abnormal impulse propagation, thus enabling reentrant electrical activity [13].

## 2. Role of Myofibroblasts in Perpetual Atrial Fibrillation

Atrial interstitial fibrosis has been shown to increase with age in humans and has been observed in patients with atrial fibrillation (AF) [14, 15], in animal models of aging [16, 17] and in congestive heart failure [18]. Through these studies, it has been shown that atrial fibrosis creates a substrate that promotes AF. Increased collagen deposition has been documented in patients with AF secondary to mitral valve disease versus those in sinus rhythm [19]. Extracellular matrix volume and composition correlate with AF persistence [20]. These findings highlight the association between atrial fibrosis and AF, although determining the causal importance of tissue fibrosis in AF occurrence and persistence remains an important challenge.

AF is also capable of enhancing atrial fibrosis. In human lone AF, it has been shown that long-term assessment of patients diagnosed with AF, which had normal sized atria upon diagnosis, does lead to structural remodeling of the atria causing atrial enlargement and dilatation over a subsequent period of 20 months [21]. The studies suggest that atrial fibrosis acts as both a trigger and a by-product of AF, potentially through a mechanism affecting signaling pathways associated with atrial dilatation [22, 23].

The mechanism of AF that is associated with an increased level of fibrosis is still under debate as both focal and reentrant mechanisms have been observed in patients and animal models of AF. In the dog model of ventricular tachypacing induced congestive heart failure, atrial fibrosis causes localized regions of conduction slowing, increasing conduction heterogeneity and providing an AF substrate [18]. Conduction abnormalities provide a basis for unidirectional conduction block and macroreentry [24].

In contrast, in the study by Stambler et al. on dogs with rapid ventricular pacing-induced congestive heart failure, AF was shown to be focal in origin caused by triggered activity [25]. This triggered activity was shown to be produced by delayed afterdepolarizations initiated by intracellular Ca^2+^ overload. Drugs that reduced intracellular Ca^2+^ levels (verapamil, flunarizine, and ryanodine) all terminated AF. Fenelon et al. expanded on this study by performing biatrial mapping in dogs with heart failure and showed that the majority of AF episodes had a focal mechanism [26].

There is evidence that atrial fibrosis is associated with a profound remodelling of the atrial pacemaker complex. It has been shown that the function of the SAN declines in AF [27], heart failure [28–30], and with age [31], conditions associated with an increased level of fibrosis. A strong correlation between these conditions and the incidence of sick sinus syndrome has been observed [31, 32]. It should be noted that, histologically, the healthy SAN is distinguished from the surrounding atrial muscle by a remarkably large amount of interstitium (e.g., up to 75%–90% of SAN volume in cat) [33]. It allows SAN electrical insulation from the surrounding atrial myocardium, except for several critical conduction pathways. Indeed, the SAN as a leading pacemaker requires both anatomical (fibrosis, fat, and blood vessels) and/or functional (paucity of connexins) barriers to protect it from the hyperpolarizing influence of the surrounding myocardium [34–36]. The presence of conduction barriers and pathways explain how a small cluster of pacemaker cells in the SAN pacemaker complex manages to depolarize widely distributed areas of the right atria. An increased level of interstitial fibrosis can further insulate the SAN thereby altering the delicate balance between depolarized cells (source) and the resting tissue ahead (sink) [37].

**Figure 1:**
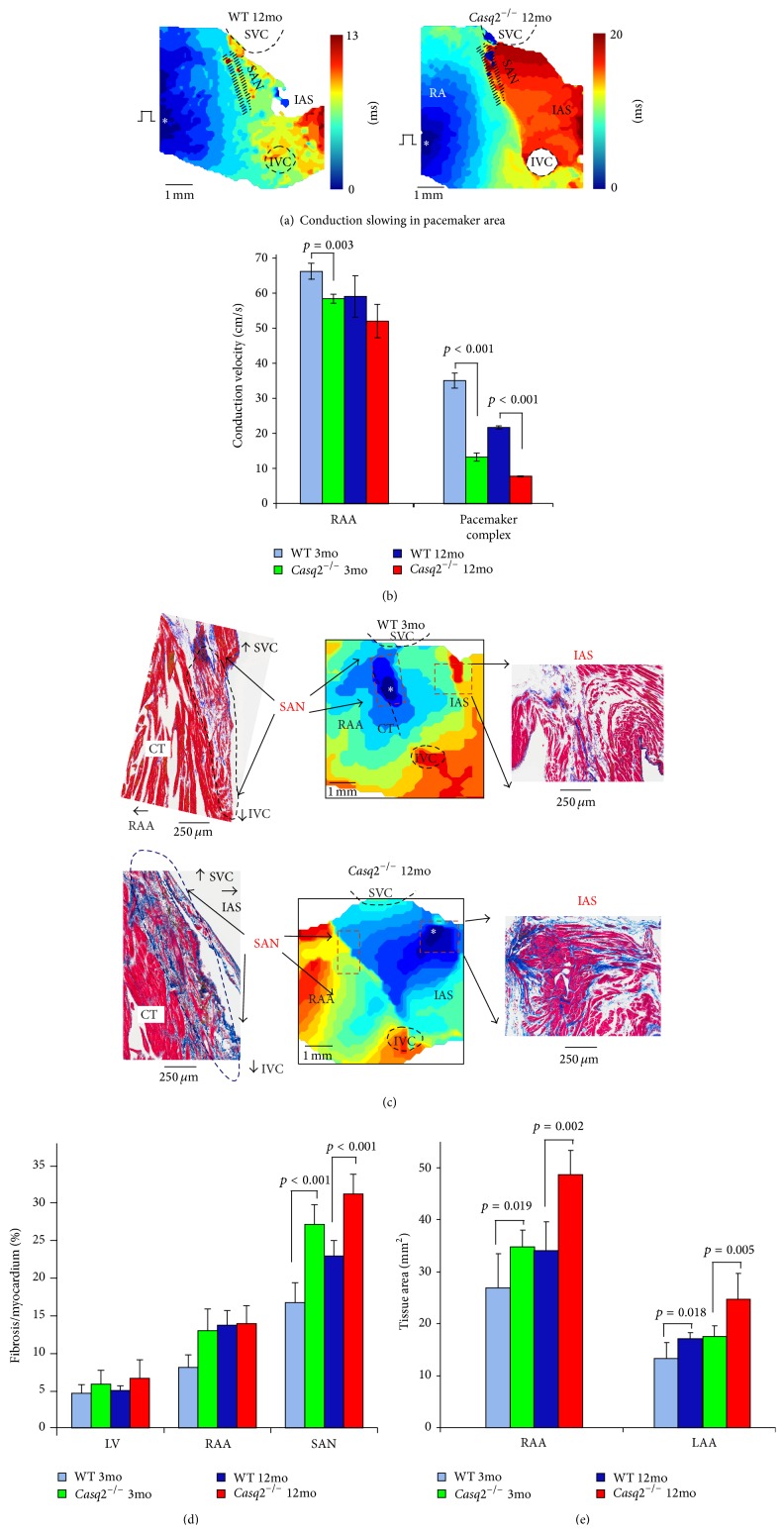
Enhanced fibrosis, sinoatrial node (SAN) conduction blocks, and atrial enlargement in *Casq*2^−/−^ hearts. (a) Representative examples of atrial activation during SAN recovery time (SANRT) measurements in 12-month old (mo) wild type (WT, left) and 12 mo *Casq*2^−/−^ (right) mice at baseline. Activation maps were obtained at continuous pacing (S1S1 = 100 ms) during SANRT measurements at baseline. SVC and IVC: superior and inferior vena cava; RAA and LAA: right and left atrial appendages; RV and LV: right and left ventricles; CT: crista terminalis; IAS: interatrial septum; AVJ: atrioventricular junction. (b) Average data for conduction velocity measured in RAA and within the pacemaker complex at S1S1 = 100 ms pacing in both 3 mo and 12 mo WT and *Casq*2^−/−^ mice. (c) Histological analysis of the atrial pacemaker complex in WT and *Casq*2^−/−^ mice is shown. Top: 3 mo WT mouse demonstrates a typical SAN activation at control. Histological staining of the same SAN preparation shown in activation maps confirms location of the SAN. Sections were cut through the SAN preparation parallel to the surface. An enlarged part of the stained preparation (marked by a red dotted rectangle on the activation map) demonstrates the compact part of the SAN (blue rectangle) separated from the atrial muscle (green rectangle) on the other side by connective tissue. Bottom: 12 mo *Casq*2^−/−^ heart demonstrates structural remodeling of the atrial pacemaker complex. (d) Average ratio of fibrotic tissue content to cardiac tissue measured in different areas in both 3 mo and 12 mo WT and *Casq*2^−/−^ mouse hearts. (e) Atrial tissue area calculated for both right and left atria in 3 mo and 12 mo WT and *Casq*2^−/−^ hearts (reprinted with permission from [4]).

**Figure 2:**
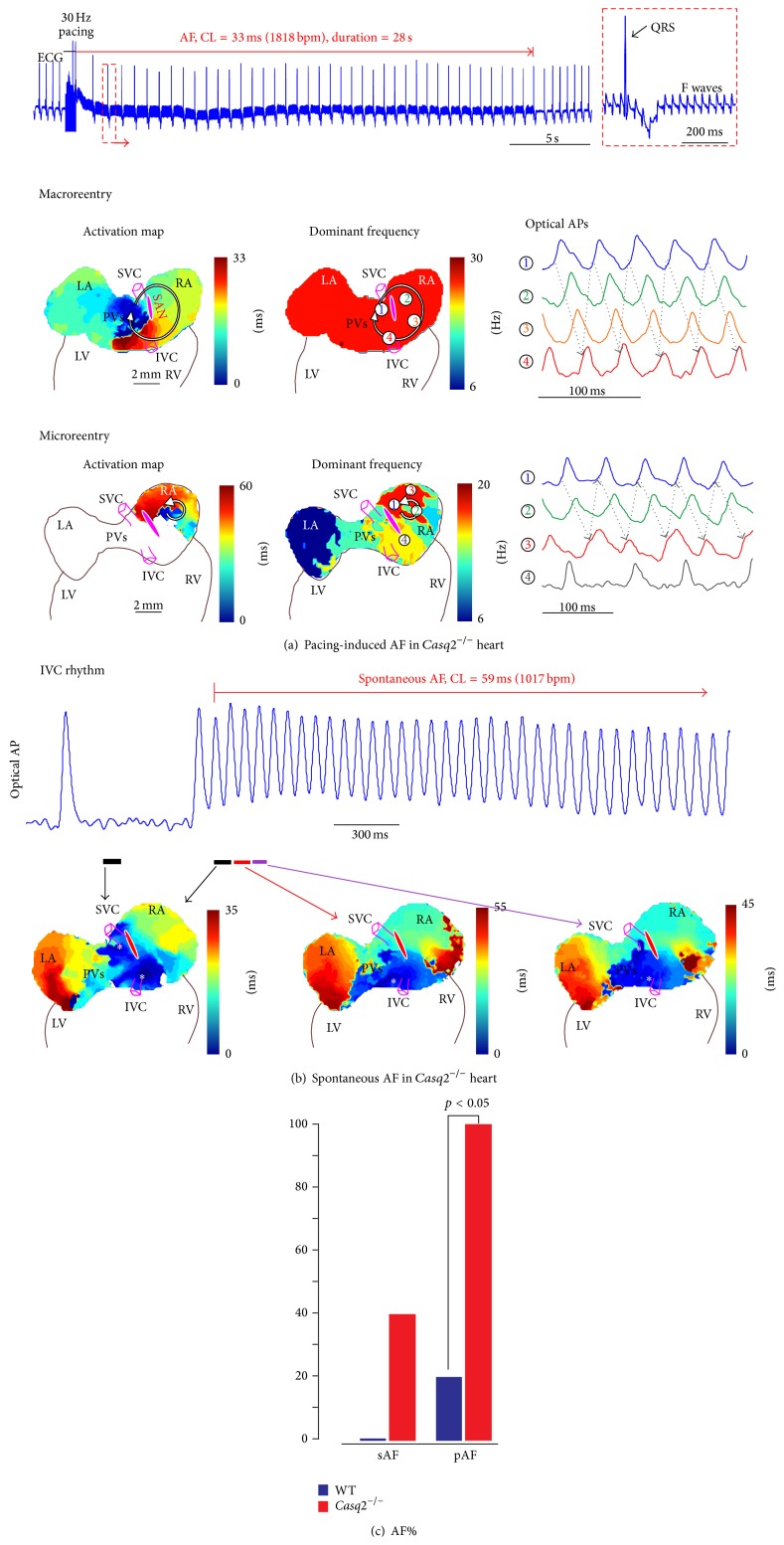
Increased susceptibility to atrial flutter/fibrillation (AF) in *Casq*2^−/−^ hearts. (a) Rapid-pacing-induced AF in *Casq*2^−/−^ hearts under isoproterenol and acetylcholine treatment. The pseudo-ECG showed that rapid pacing at 30 Hz induced AF which lasted for 28 seconds. The regular fibrillatory (F) waves indicating atrial flutter is zoomed in on the right. Both macro- and microreentry were drivers for the AF in *Casq*2^−/−^ hearts. The reentrant circuits are shown in the activation maps on the left; maps in the middle show the dominant frequency (or the reciprocal of the averaged cycle length) at various locations. During macroreentry, dominant frequency was uniform. In contrast, during microreentry, dominant frequency was locally higher in the microreentry circuit area. On the right are the sample optical action potentials (OAPs), whose locations were marked by numbers in the frequency maps. Abbreviations are the same as those in Figure 1. (b) Spontaneous AF occurred under Isoproterenol (Iso) and acetylcholine (ACh) treatment. As shown in OAPs and activation maps, the heart was under slow stable IVC rhythm, and then one of the IVC beats triggered a rapid burst of atrial activity. Representative OAP trace from the IVC region is shown. (c) Percentage of animals with AF inducibility in the control and *Casq*2^−/−^ groups (sAF: spontaneous AF; pAF: pacing-induced AF) (reprinted with permission from [4]).

On the other hand, an increased fibrosis can unmask the latent pacemakers by forming specialized, isolated clusters of pacemakers located throughout the atrial pacemaker complex [4, 38]. It has been known for over a century that pacemaker cells are widely distributed throughout the entire region located between the superior and inferior vena cava and between the crista terminalis and intra-atrial septum [33, 39]. Canine and human studies [40–44] have revealed an extensive distributed system of atrial pacemakers, the atrial pacemaker complex, which extends well beyond the anatomically defined SAN and includes primary and subsidiary pacemakers located within the right atrium. Functional anatomy of the atrial pacemaker complex has been extensively studied in mouse models of sick sinus syndrome. Recently, in calsequestrin 2 null mice which were characterized by an increased susceptibility to AF, we have shown, using a high-resolution optical mapping and 3D atrial immunohistology a selective interstitial fibrosis in the atrial pacemaker complex [4], Figure 1. Deletion of calsequestrin 2 depressed primary SAN activity and conduction, but enhanced atrial ectopic activity and AF associated with macro- and micro-reentry during autonomic stimulation (Figure 2). It depressed primary SAN activity and conduction but enhanced atrial ectopic activity and AF associated with macro- and microreentry during autonomic stimulation (Figure 2). Thus, the latent pacemakers will be more stable compared to the primary pacemaker, SAN, probably due to protective electrical insulation role of fibrosis. Such aberrantly isolated clusters of latent pacemakers could become activated and take over the role of the leading pacemaker which can be exaggerated during the abnormal response to autonomic stimulation.

Similar results have been observed in other genetically engineered mouse models. Deletion of some structural proteins (such as Cx40 [45, 46], ankyrin-B [38], liver kinase B1 (LKB1) [47], natriuretic peptide receptor C [48], or overexpression of tumor necrosis factor- (TNF-) *α* [49]) has been linked to enhanced fibrosis, depression of the SAN function, and increased atrial arrhythmogenesis. Shift of the leading pacemaker outside of the SAN structure and a beat-to-beat competition between different pacemakers have been revealed in these mouse models and resulted in heart rate irregularities, tachy-brady arrhythmias, and AF. Interestingly, autonomic stimulation [4] or consecutive thermal ablation of such ectopic sites [45] resulted in leading pacemaker shift back to the SAN but at a prolonged intrinsic cycle length.

In addition to the concept that enhanced interstitial fibrosis contributes to cardiac arrhythmias “indirectly” by affecting passive properties of impulse conduction, recent studies demonstrate that at least a paracrine interaction, or likely a direct electrical coupling, exists between the cardiomyocytes and (myo)fibroblasts (MFBs, see paragraphs below). It has been suggested that structural remodelling including fibrosis of the SAN complex could be attributable to abnormal Ca^2+^ handling in the pacemaker cells [28]. Enhanced diastolic Ca^2+^ could directly lead to increased fibrosis within the SAN complex as well as in the latent pacemaker areas by favouring downstream activation of apoptosis due to cytosolic Ca^2+^ overload. In fact, it has been suggested that chronic Ca^2+^ leak from the sarcoplasmic reticulum can directly stimulate cell damage and fibrogenesis [50]. Increased intracellular [Ca^2+^] could also stimulate activation of the multifunctional Ca^2+^/calmodulin-dependent protein kinase II (CaMKII) which in turn promotes myocardial dysfunction [51] and heart failure [52], SAN cell apoptosis, increased fibrosis and alternating atrial arrhythmogenesis [28].

Finally, the emerging results demonstrate that MFBs* in vitro* also promote cardiac arrhythmogenesis due to direct implications upon cardiomyocyte electrophysiology [9, 53]. When coupled to cardiomyocytes, MFBs have a depolarizing effect on cardiomyocyte resting membrane potential, which can lead to partial or total sodium channel inactivation. Recent studies have indicated that depolarization in the resting membrane potential of fibroblasts is the most critical factor promoting cardiomyocyte early after depolarizations ectopic activity [8, 54].

## 3. Role of Myofibroblasts in the Heart

Under the pathological conditions like hypertension, fibrosis, and infarction, MFBs appear in the myocardium. These cells have an important role in reparative fibrosis; they share a phenotype with fibroblasts and smooth-muscle cells and were first identified years ago in skin wound tissue [55] and granulation tissue [56]. However, it is not yet known if these cells are resident changed-phenotype-fibroblasts, endothelial-mesenchymal derived cells or from fibrocytes [57, 58]. Their role is merely reparative and they disappear following programmed cell death. Currently, the most reliable marker for MFBs is alpha-smooth-muscle actin (*α*SMA), which is expressed in smooth-muscle cells but not in fibroblasts. It has also been shown that MFBs participate in the process of reparative fibrosis in the lung [59], liver [60], and pancreas [61], where they produce excessive ECM, a process similar to that of fibrotic heart remodelling. The trigger for recruitments of MFBs to the diseased heart is not fully understood. The local upregulation of cytokines including foremost TGF-B1 seems to play a prominent role. However it has been demonstrated that tissue stiffening following excessive ECM deposition drives transdifferentiation of precursor cells into forming fibrogenic MFBs [62]. Myofibroblasts themselves produce uncontrolled ECM; hence, a vicious circle ensues. It has also been shown that variation in oxygen (O_2_) concentration plays a key role in the proliferation of cardiac MFBs. Adult mouse cardiac fibroblasts cultured at 21% O_2_ express* de novoα*SMA [63]; in contrast the same was observed when human fetal cardiac fibroblasts were exposed to low percentage O_2_ [64].

## 4. Electrical Communications between MFBs and Adjacent Parenchymal Cells

MFBs form gap junctions with the resident parenchymal cells and can exist in different organs like skin [65], intestines [66], and bladder walls [67]. In the healthy heart, MFBs are present only in the valve leaflets; postinfarct MFBs appear in large numbers a few days after injury at the site of infarction. These MFBs differ from those in skin wounds as they can persist in the infarct area for 20 years [68, 69] whilst maintaining intimate contact with the surviving cardiomyocytes.

**Figure 3:**
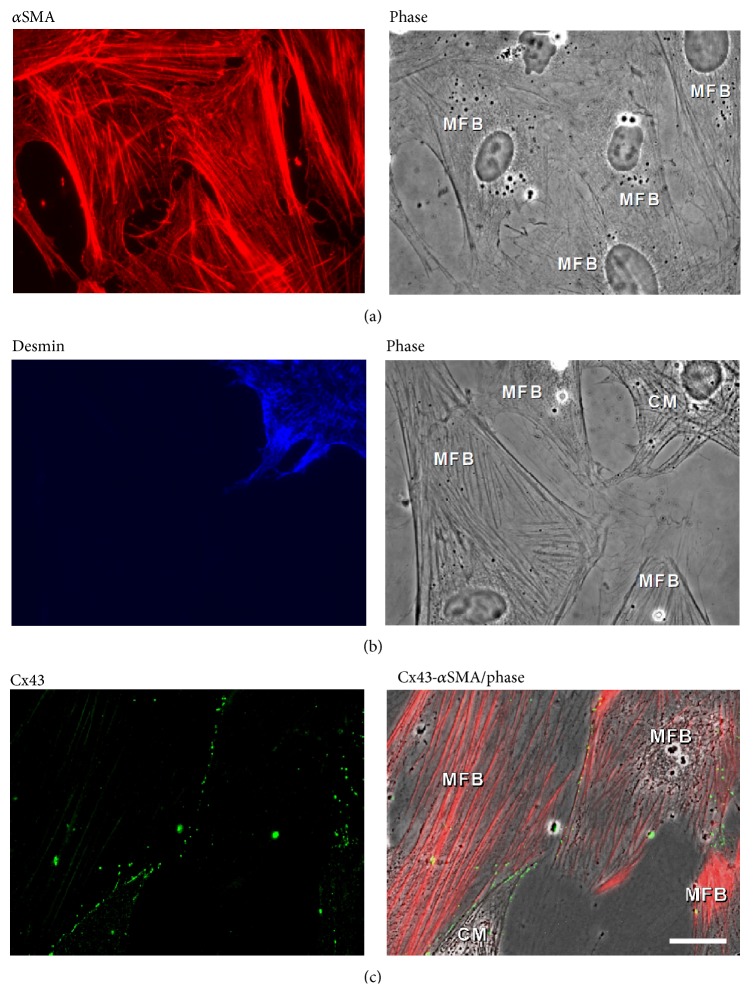
Phenotype characteristics and electrical coupling between myofibroblasts and cardiomyocytes. (a) Immunocytochemistry and phase contrast picture shows expression of *α*SMA (red) in cardiac fibroblasts, which have differentiated to myofibroblasts after 3 days in culture. (b) Parenchymal cardiomyocytes and not stromal myofibroblasts express desmin (blue). (c) Myofibroblast *α*SMA positive cells (red) express Cx43 (green) at cell-cell contacts and at contacts with cardiomyocytes. The corresponding phase contrast picture shows spatially the contact between coculture of myofibroblasts and cardiomyocytes (reprinted with permission from [5]).

**Figure 4:**
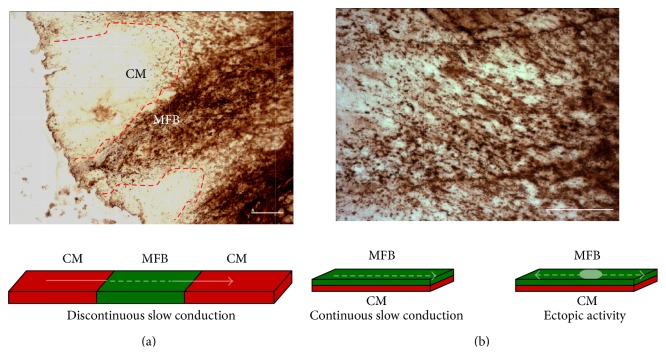
Characteristics of heterocellular interaction between myofibroblasts and cardiomyocytes in regionally infarcted rat heart. (a) Top. Immunohistochemistry picture of rat heart slices after 37 weeks of coronary occlusion. A region of myofibroblasts (brown) stained for *α*SMA physically separates two bundles of cardiac myocytes. Bottom. Schematic representation of heterocellular culture mimics the* in vivo* situation. (b) Same as A with a MFBs stratum infiltrated into cardiac tissue (top) with schematic represented situation (bottom) (with courtesy: Dr. Alex Lyon, NHLI, Imperial College, London. Unpublished).

It is assumed that MFBs primarily differentiate from resident fibroblasts. This process is initiated by transforming growth factor *β* (TGF*β*), followed by an activation of several “canonical” cellular pathways (Smad, ERK, P38 kinase, AP-1 but not JNK) [59]. In culture, neonatal rat cardiac fibroblasts undergo transdifferentiation into MFBs.* De novo* expression of *α*SMA increases in parallel with the expression of connexin43 (Cx43) [70]; thus, cutback in expression of Cx43 by small interfering RNAs technique significantly inhibits *α*SMA expression. There is evidence that fibroblasts in the infarct scar tissue express Cx43 and Cx45 [71]. Other investigations have demonstrated that these fibroblasts are in fact (myo) fibroblasts (Figure 4) [72]. However, questions about electrical coupling between MFBs and cardiomyocytes remain unanswered. It is essential to note that it is not yet reported whether MFBs* in vivo* establish a heterocellular electronic coupling with cardiomyocytes. We have successfully engineered the heterocellular contact* in vitro* by coculturing neonatal rat cardiomyocytes and MFBs from cardiac origin (Figure 3). As expected [73], these fibroblasts became MFBs when cultured on rigid substrates (glass coverslips). This was confirmed by observed* de novo* expression of *α*SMA (Figure 3(a)). We have also demonstrated that* in vitro* MFBs express gap junction proteins Cx43 and 45 (not Cx40) at MFB-MFB cell-cell contacts and importantly also at MFB-CM cell-cell contacts (Figure 3(c)) [7]. The successful establishment of this heterocellular contact* in vitro* together with our previous investigations into a variety of histoarchitectures* in vivo* now allows for the study of two different situations normally encountered in the infarcted heart.

Immunohistochemical images of chronic infarct in rat cardiac tissue (37 weeks) shown in Figure 4 demonstrate that there is an intimate contact between the areas heavy populated with MFBs (*α*SMA, brown) and the areas of surviving cardiomyocytes (white). We hypothesised that an area of MFBs might (i) interrupt or affect impulse propagation and (ii) induce ectopic activity, due to electrical coupling.

## 5. Areas of Myofibroblasts Linking Up Separate Bundles of Cardiomyocytes

The general assumption is that cardiac impulse conduction is blocked at site where cardiomyocyte areas are in contact with collagenous septa or with fibrotic tissue (like infarct regions or sutures site follow heart transplantation). This latter case sporadically reports an unexpected synchronization between donor and recipient heart [74]. Because MFBs are present in the fibrotic tissue, we tested this hypothesis, by engineering the situation represented in Figure 4(a) top,* in vitro*, by seeding cardiomyocytes in a geometrical defined pattern, and interrupted them with an insert of MFBs (Figure 4(a), bottom).

**Figure 5:**
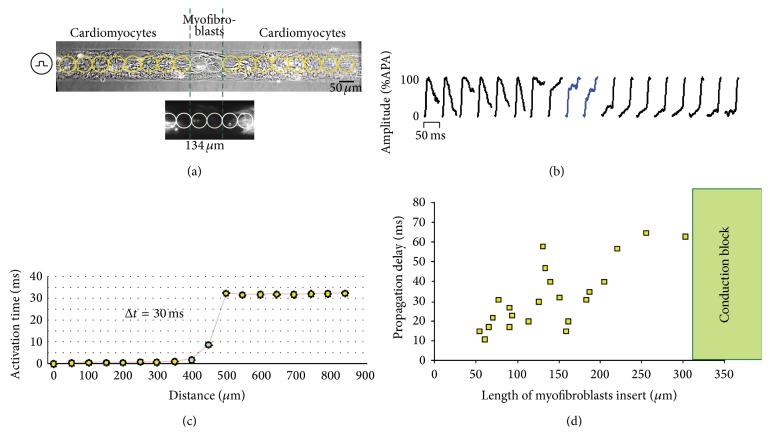
Myofibroblasts act as passive electrical paths for impulse conduction. (a) Heterocellular construct of a left-stimulated strand of cardiomyocytes (top) interrupt by a pure region (length = 134 *μ*m) of myomesin-deficient myofibroblasts (bottom). Circles indicate the optical mapped area detected from each photodetector. (b) Optical action potential upstrokes recorded in a detected length of ~50 *μ*m. (c) Activation times reconstruct from each action potential upstrokes; in both cardiomyocytes areas, the activation is almost immediate whereas the propagation into myofibroblast area exhibits a delay of 30 ms. (d) Summary of propagated delays related to the inserts' length, therefore indicating a passive electronic transmission of up to ~320 *μ*m (modified with permission from [6]).

Details of the experiments are represented in Figure 5(a) [6]. Strands were stimulated from the left hand side and the characteristics of impulse propagation were assessed optically after being exposed to a voltage sensitive dye [75]. For the final analysis, we took into consideration only the insert of MFBs without “cardiomyocytes contamination” (Figure 5(a), lower panel). Each photodiode recorded an optical action potential upstroke, which was correlated with the activation time. Whereas activation time was rapid in the cardiomyocyte area, a passive local electrotonic transmission induced a delay of 30 ms across the MFB insert (Figure 5(c)). As shown in Figure 5(d), the delay is strictly related to the length of the insert. Under these experimental conditions MFBs can support impulse propagation up to ~320 *μ*m; at lengths greater than this, propagation invariably failed. These experiments demonstrate that the heterocellular electrical coupling between the two cell populations can reinstate conduction across an interrupted network of cardiomyocytes resulting in a discontinuity of propagation. In consequence, one has to take into consideration that patchy fibrosis as encountered in the remodelled atria (i.e., ageing) [76] may alter the normal pattern of propagation by inducing discontinuous slow conduction, playing a key role in the context of reentrant circuits.

## 6. Myofibroblasts Overlaid as to Completely Cover an Area of Cardiomyocytes

**Figure 6:**
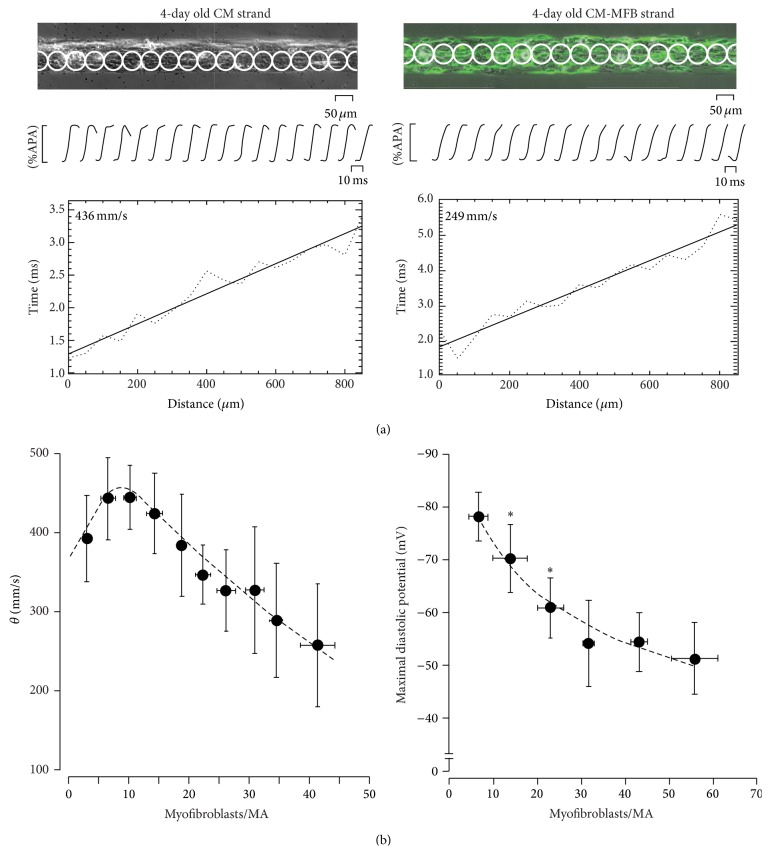
Impulse conduction in myofibroblasts coated cardiomyocyte strands. (a) Same as Figure 3(a) for optical action potential recording in a pure (left) and *α*SMA positive myofibroblasts (green) coated cardiomyocyte strand (right). Heterocellular construct shows a reduced conduction velocity (249 mm/s) compared to control (436 mm/s, *p* < 0.001). (b) Overall analysis of conduction velocity and maximal diastolic potential related to MFBs density calculated as number of cells per measurement area. Left: conduction velocity denotes biphasic behaviour due to the supernormal sodium based conduction follow gradual depolarization that occurs from the increment of myofibroblasts density. Right: myofibroblasts reduce maximal diastolic potential in a cell density-dependent manner (modified with permission from [7]).

To further investigate any direct electrical coupling consequences, MFBs were also cultured in order to overlay the cardiomyocytes. In Figure 4(b), the situation is such that the fibrotic area is heavily populated with MFBs, which infiltrate and diffuse throughout the area of cardiomyocytes, thus increasing the heterotissue interaction (as compared with Figure 4(a)). Due to the depolarized resting membrane potential of MFBs [7], we hypothesized that these circumstances, together with the increased electrical cell-to-cell interaction area, might produce a large depolarized region which can affect local impulse propagation. This, in turn, might reduce the conduction velocity for the cardiac tissue, which is in contact with MFBs. Utilising the same pattern growth technique we engineered 80 *μ*m wide strands of neonatal rat ventricular cardiomyocytes, to which a layer of MFBs is seeded on top of [7] (Figure 4(b), bottom). The conduction velocity (*θ*) in control preparations, which are virtually devoid of MFBs, is high (~43 cm/s, Figure 6(a), left). However, the presence of MFBs drastically reduces *θ* by up to 25 cm/s (Figure 6(a), right). An overall analysis of *θ* dependence on MFB coverage area is represented in Figure 6(b) left; *θ* denotes biphasic behaviour towards the number of MFBs per measured area. This behaviour is highly reminiscent of the phenomena of supernormal conduction in cardiac tissue investigated both* in vivo* [77] and* in vitro* [78]. Both demonstrated that *θ* is biphasically dependent on the gradual increment of extracellular potassium concentration. Similar, but in a MFBs density-dependent manner, our results show a similar behaviour, suggesting that MFBs may directly depolarize cardiomyocytes resembling the well-known depolarizing effect of [K^+^]_out_. This hypothesis was proved by conventional intracellular microelectrode techniques for measuring diastolic resting membrane potential (*V*_*m*_). We found that MFBs gradually depolarized cardiomyocytes in a density-dependent manner (Figure 6(b), right) where recorded *V*_*m*_ dropped from ~−80 mV at a MFB density less than 5% to ~−55 mV when more than 40% of the cardiomyocytes area is covered by MFBs. This data propose that, assuming the same effect* in vivo* at the epicardial border zone where the minimal wall thickness is comparable to a two-dimensional layer, infiltrated laminae of MFBs might induce epicardium slow conduction velocity. In contrast, in a 3D architecture (ventricular wall) the coupling of cardiomyocytes bordering infarct area might counterbalance this depolarizing effect. The consequences in the context of AF are clear: MFBs can directly depolarize the surrounding cardiomyocytes tissue and thus lead to local conduction slowing and enhance the likelihood of arrhythmia (cf. paragraph below).

## 7. Myofibroblasts Induce Ectopic Activity in Cardiac Tissue

**Figure 7:**
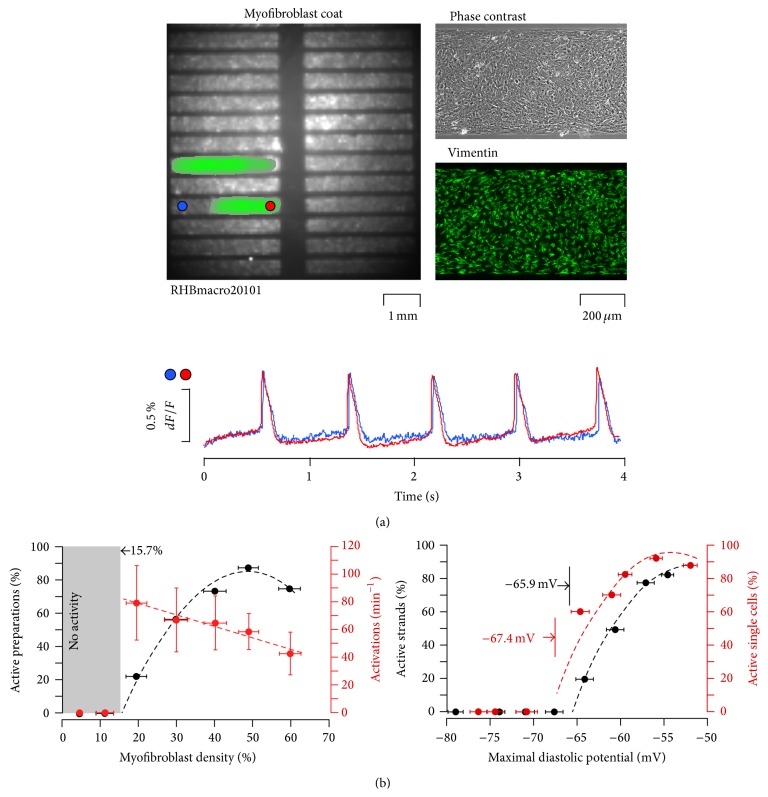
Myofibroblasts induce ectopic activity. (a) Upper row: overview of a preparation consisted of 24 myofibroblasts coated with cardiomyocyte strands (0.6 × 4.5 mm). Right: details of the microarchitecture (a phase contrast photo) and of the MFB cover layer (vimentin immunostaining) of an individual strand. Lower row: propagated action potential recorded optically at specific sites (blue and red circles in the overview). (b) Left: ectopic spontaneously active strands and spontaneous frequency correlate with myofibroblast density. Spontaneous activity is invariably absent when myofibroblast density is less than 15.7% of the total area examined. Right: electrical spontaneous activity correlated with maximal diastolic potential. Similar to active strands where membrane potential threshold for elicit automaticity is −65.9 mV, single cardiomyocytes elicit spontaneous action potentials at −67.4 mV (modified with permission from [8]).

The last part of the study sought to investigate the intimate contact between cardiomyocytes and MFBs (Figure 4(b), right) and how ectopic electrical activity was elicited following heterocellular coupling [8]. An* in vitro* fibrotic situation was created by coating strands of neonatal rat ventricular myocytes with increasing densities of MFBs (Figure 7). Spontaneous electrical activities were recorded for 4 seconds. The overview of preparation in Figure 7(a) corresponds to a single frame taken from such recording. In this point in time the quasicompletely coverage of MFB elicits spontaneous activity in two strands; the recording shows action potentials occurred regularly with a frequency of 75 bpm. Crucially, the presence of spontaneous activity is strictly proportional to MFBs densities (Figure 7, top). When MFB coverage was below 16% all the preparations were invariably quiescent; however, when coverage was above ~80%, all preparations exhibited spontaneous activity. In contrast, we found beat frequency reduced from ~80 bpm (20% MFBs density) to ~40 bpm (60% MFBs density) due to progressive reduction in the diastolic membrane potential (Figure 6(b)). A comparison of cardiac membrane potential between isolated cardiomyocytes and heterocellular strands is demonstrated in Figure 7(b), right. Gradual reduction of resting *V*_*m*_ in single cardiomyocytes was examined using a patch clamp technique. Stepwise depolarization during injection of 30 sec long current pulses exhibits electrical spontaneous activity elicited at membrane potential less negative than −67 mV. These results were similar to those found in the heterocellular strands where spontaneous impulse initiation was induced with a minimal density of MFBs corresponding to ~−66 mV. These findings indicate that the heterocellular coupling between MFBs and cardiomyocytes might structurally form an ectopic focus. The firing area is preferentially generated from MFBs and not from the injured cardiomyocytes. In our experiments, the cardiac network appears healthy but ectopic activity could be as well induced.

## 8. Is the Myofibroblast a Possible Target for Atrial Fibrillation?

**Figure 8:**
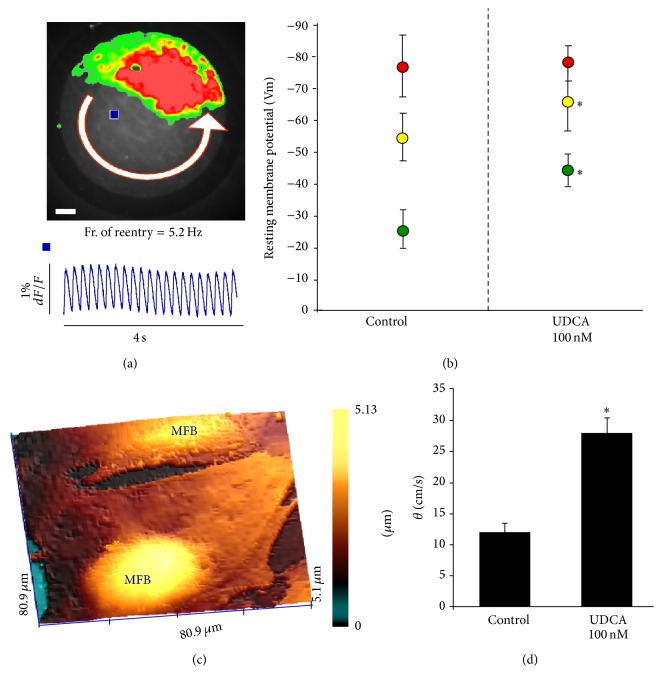
Myofibroblast as a possible cell target for antiarrhythmic therapy. (a) Reentrant excitation on myofibroblasts coated cardiomyocytes monolayer. Top: colour coded reentrant propagation. Bottom optical action potential traces. (b) UDCA hyperpolarizes MFBs membrane potential only. Red circles: cardiomyocytes monolayers. Yellow circles: heterocellular monolayers. Green circles: MFB monolayers. *p* < 0.05. (c) Topographical images obtained by scanning ion conductance microscopy of a MFB embedded in a monolayer. (d) Effect of UDCA on impulse propagation velocity in myofibroblast coated with cardiomyocytes strands (modified with permission from [9]).

Recently we have investigated the possibility to target MFB in order to suppress electrical disorders in the heart. Thanks to the collaboration with Professor Gorelik and Professor Williamson at Imperial College London, we have discovered that MFBs transiently appear during heart development and they can be responsible for fetal arrhythmias [79]. Clinically, it has been associated with a pregnant disease called intrahepatic cholestasis and the prognosis ameliorates after administration of ursodeoxycholic acid (UDCA). Because MFBs tend to depolarize the coupled cardiomyocytes, we sought to investigate whether UDCA may directly target MFBs. We found that UDCA hyperpolarizes MFB membrane potential by targeting the sulphonylurea receptor of  I_K1_ channel [9], reestablishing the normal conduction velocity and terminating reentrant arrhythmia (Figure 8). A double-blind randomized placebo-controlled crossover trial is under investigation by administering UDCA in patient with chronic heart failure [80].

## 9. Outlook

**Figure 9:**
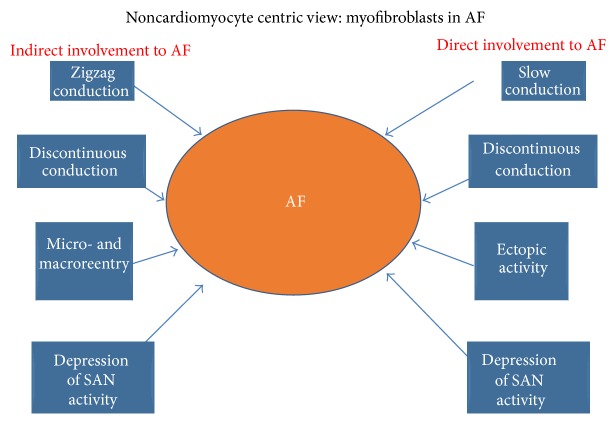
Schematic representation of a “well-known” (indirect) and “proposed” (direct) involvement of myofibroblasts in atrial fibrillation (AF).

Electrical communication between the stromal and parenchyma tissue has been the focus of much research over the last 50 years. Certain “myths,” like that we are using a total of 10% of our brain, have been dispelled, including the discovery that indeed only 10% of the human brain is made of neurons, with the rest comprised of “Glia,” identified as stromal “glue” or nerve “putty,” which merely fills the spaces within the parenchyma tissue. In the last two decades researchers have shown that Glia cells express gap junctions and interact directly with neurons [81, 82].* In vitro*, stromal cardiac MFBs are electrically and mechanically coupled with cardiomyocytes and this pairing disturbs the electrical homeostasis of the parenchymal cardiac tissue [83, 84]. If the same situation in pathological cardiac tissue will be observed* in vivo*, MFBs could be considered as a new cellular target for cardiac arrhythmia (Figure 9). Strategies might focus on (i) inducing the MFBs “inactive” (overturn the phenotype back to fibroblast and thus circumvent the electrical coupling) or (ii) targeting these cells, pharmacologically or genetically, for radically hyperpolarization [9, 85]. Evidence obtained so far requires further characterization in order to fully understand the impact(s) of heterocellular interactions upon the complex 3D remodelling of the cytoarchitecture, which occurs during heart failure.

Interestingly, there is speculation that MFBs might appear transiently during heart development and follow the partial state of fetal hypoxia [64] and in the aged heart [76, 86]. Additional studies are necessary to understand if MFBs are not only proarrhythmogenic in heart failure but also during heart development and aging; these are frequently subjected to other pathological “MFB-triggering” situations (diabetes, autoimmune disorder, and metabolic diseases).

Problems regarding engraftment for tissue regeneration have also been investigated and reveal that if other cells, less polarised than cardiomyocytes, were to form gap junctions with cardiomyocytes, spontaneous activity could be induced [87]. This was highlighted by our study where coating cardiomyocytes with Cx43-transfected-HeLa cells ([8], data not shown) give rise to spontaneous activity. Further studies are necessary in order to understand and predict an accurate arrhythmogenic mechanism following cell engraftment in heart failure models using possibly or conductive patches [88], embryonic cardiomyocytes [89], and progenitor stem cells [90]. Regarding the cell therapy, it is also unclear whether engraftment will perturb the electrical homeostasis of cardiac tissue due to an intrinsic resting membrane potential, and there exists the possibility that the engrafted cells might electrically couple with MFBs. Both of these may cause the regenerated cardiac tissue engraftment to become an unexpected source of arrhythmia.
